# Dr. William Taft. Jr.

**Published:** 1901-12-15

**Authors:** 


### OBITUARY.

### DR. WILLIAM TAFT, JR.

Word has just come by cable message from Dr. Ottofy, of Manila, that Dr. Will Taft, son of Dr. Wm. Taft and grandson of Dr. Jonathan Taft, had died in Manila, where he went a few months since to engage in the practice of dentistry with Dr. Ottofy. It is not known as yet what the cause of his death may have been. He was a young man of good health and had promise of making a good reputation for himself as a pioneer in teaching the people of our new responsibilities the care of the teeth in a wholesome manner. The sympathy of the profession will be most cordially extended to the father and grandfather.

